# Materials modelling in the University of Limpopo

**DOI:** 10.1098/rsfs.2024.0005

**Published:** 2024-08-09

**Authors:** Phuti E. Ngoepe, Alan V. Chadwick, Happy M. Sithole, Khotso D. K. Mokhele, C. Richard A. Catlow

**Affiliations:** ^1^ Materials Modelling Centre, University of Limpopo, Private Bag x1106, Sovenga 0727, South Africa; ^2^ School of Physical Sciences, University of Kent, Canterbury CT2 7NZ, UK; ^3^ The Center for High Performance Computing, Council for Scientific and Industrial Research, Meiring Naude Street, Brummeria 0001, South Africa; ^4^ Hans Merensky Holdings, Saxonworld, Johannesburg 2132, South Africa; ^5^ Department of Chemistry, University College London, 20 Gordon Street, London WC1H 0AJ, UK; ^6^ School of Chemistry, Cardiff University, Park Place, Cardiff CF10 3AT, UK

**Keywords:** South Africa, computational modelling, materials, National Research Foundation, Royal Society

## Abstract

This article provides insights into building research capacity in computational modelling of materials at the University of Limpopo (UL), formerly University of the North, in South Africa, through a collaboration with a consortium of universities in the United Kingdom (UK) through the support of the National Research Foundation (NRF), formerly the Foundation for Research and Development, and the Royal Society (RS). A background that led to the choice of building research capacity at historically disadvantaged universities in South Africa, including the UL, is given. The *modus operandi* of the collaboration between the UL and several UK universities on computational modelling of materials is outlined, together with the scientific highlights that were achieved in themes of minerals, energy storage and alloy development. The capacity built in terms of human capital and institutions set up is shared, which is followed by a discussion of the continuing research activities after the formal NRF–RS collaboration ceased with more alignment to industrial applications with national and international support. We conclude by highlighting the success of the project in capacity-building and consolidating the Materials Modelling Centre with developments of high-performance computing in South Africa and the African continent. We comment on the lessons learned regarding successful capacity-building programmes.

## 1. Introduction

Computational modelling at the atomic and molecular levels is now an integral component of contemporary materials chemistry, physics and engineering [1]. It is used routinely and predictively in studies of structure, surface and defect, properties of materials as well as in modelling sorption, diffusion and reactivity and in understanding nucleation and growth. The techniques are also increasingly applied to low-dimensional and nanostructures. In the mid-1990s, a collaboration was initiated between the group of Professor Phuti Ngoepe in the (then) University of the North (UNIN; now, University of Limpopo, UL) and a consortium of United Kingdom (UK) materials modelling scientists, with the aims of both establishing a strong collaborative programme between the UK and South African scientists in the field of materials modelling and developing a centre of excellence at UNIN, whose programme would have a strong focus on areas of key importance to the economic and societal needs of South Africa.

Both the collaboration and the centre developed rapidly. The scientific programme had a strong focus on materials for *energy technologies*, especially, high-energy-density batteries, on *alloys* and on processes and materials relating to *mineral extraction technologies*. The collaboration was fostered by frequent exchanges of staff and students between the growing centre and UK universities, by collaborations with South African industry and by a stimulating annual conference, while continuing support from the university and South African funding agencies supported the expansion of the centre. The centre became increasingly productive in terms both of scientific output and of staff trained in the latest developments in the field. The Materials Modelling Centre (MMC) is now a successful self-sustaining institution working at the forefront of the field, with continuing collaborations with the UK and other international partners.

In this article, we will trace the origins of the collaboration, describe how the collaboration then developed and worked, review the outputs and achievements and discuss the present programme and priorities. We will conclude with some general comments and reflections on the programme and the centre, and we hope to show how modest capacity-building programmes can contribute effectively to both training and institutional strengthening and produce high-quality science; but perhaps, most importantly, it can have a long-term impact.

## 2. Origins

### 2.1. The historical context

All nations have special dates on which transformational events took place that changed the course of their histories. For South Africa, 2 February 1990 is one such date. On this date, the then president of apartheid South Africa, F. W. De Klerk, made a dramatic speech in parliament in which he announced that all political prisoners were to be released, and the banning of all political parties and liberation organizations was lifted. Just like that, Nelson Mandela and the leaders of the various political and liberation struggle organizations were to go free. These dramatic announcements heralded the start of a brand new project of national deconstruction and reconstruction.

With the United Nations having declared apartheid a crime against humanity, apartheid South Africa had virtually become a pariah state, shunned by most nations in the world and barred from membership and participation in many international and inter-governmental organizations. The announcements made on 2 February 1990 unlocked tremendous goodwill from across the world with pledges of assistance and partnerships in the reconstruction of the new democratic South Africa. The African National Congress gained a landslide victory in the first democratic election held on 27 April 1994, and Nelson Mandela became the first President of the democratic South Africa.

The inauguration of the new president became a global affair, with world leaders and all sorts of dignitaries descending upon the country to be part of this seminal moment of the liberation of the last remaining colonized African country. The UK prime minister at the time, Mr John Major, was among the attendees, bringing with him a British Airways Boeing 747 full of officials and dignitaries to engage with all spheres of development challenges with offers of assistance and partnerships. Included among these was a scientific delegation led by the then Science Advisor to the UK Prime Minister, Sir William Stewart and the then President of the Royal Society (RS) of London, Sir Michael Attiyah.

To understand the magnitude of the project to deconstruct and reconstruct the science and technology system of South Africa, one has to refer to a statement made in parliament in 1955 by the architect of apartheid, Hendrik Verwoerd, who became prime minister from 1958 to 1966. He stated as follows: ‘What is the use of teaching the Bantu child mathematics when it cannot use it in practice? That is quite absurd. Education must train people in accordance with their opportunities in life, according to the sphere in which they live’. The democratic South Africa faced a gargantuan challenge of mitigating the damage caused by the Verwoerdian dogma, replacing it with an educational system that brought the majority Black population into the mainstream of global educational and science enterprise.

The erstwhile Foundation for Research Development (FRD), which is the predecessor to the current National Research Foundation (NRF), was the premier state-funded agency to fund higher education research in the natural sciences, engineering and technology. As an agency created in the apartheid state, the FRD patterns of funding research had been overwhelmingly biased towards Historically White Universities (HWUs), almost to the total exclusion of those universities that had been designated by the apartheid state to educate the majority Black students. The mandate of the FRD thus had to pivot in 1994 to service equally all South African higher education institutions and to do so in full recognition of the huge gaps that had been created by its own historical discriminatory funding patterns. The FRD’s response was to design a new ring-fenced University Development Programme (UDP) earmarked for Historically Black Universities (HBU).

### 2.2. The response

The delegations accompanying the UK prime minister to the inauguration of President Mandela engaged with various sectors to seek areas of possible assistance and collaboration. Sir William Stewart and Sir Michael Attiyah thus visited the FRD. Sir Michael Attiyah invariably focused on the contribution that the RS could make and the FRD responded with a rather audacious proposal. What if the excellence that defines the membership of the RS could be deployed in the developmental research initiatives of the UDP? This approach, the FRD argued, goes beyond just mere facilitation of the research collaboration between South Africa and the UK, which, for historical discriminatory reasons, will favour the White South African scientific community and historically White institutions. The approach will bring the excellence of the UK science enterprise into the guts of the transformational project that was at the centre of the mandate of the new democratic state.

The proposed concept was to work as follows: the FRD identified potential principal investigators (PIs), in five HBUs that already had credible research credentials, who could become lead partners in the collaboration, with matching counterparts who would be selected by the RS. The areas of collaboration were agreed as follows: physics/material sciences at the UNIN (now UL) with Professor Phuti Ngoepe as the PI; biotechnology/microbiology at the University of Durban Westville (now merged into the University of KwaZulu-Natal) with Professors Bala Pillay and Gansen Pillay as the co-PIs; molecular biology/bioinformatics at the University of the Western Cape (UWC) with Professor Jasper Reese as the PI; inorganic chemistry at the University of Zululand with Professor Mack Zulu as the PI; and pasture/range science at the University of Fort Hare (UFH) with Professors Trollip and Jan Raats as the PIs.

Once the matching of the counterparts has been agreed, the FRD and the RS would jointly fund planning meetings between the pairs of counterparts to deliberate on possible research projects that they could collaborate on. Successful planning meetings would generate research proposals that would indicate costs incurred in South Africa, which would be covered by the FRD, and costs in the UK, which would be covered through UK funding mechanisms.

The announcement of the initiative was not received with universal acclaim in South Africa, particularly by the HWUs. There were some who questioned the appropriateness of burdening the scientific excellence of the membership of the RS with developmental challenges. To them, an excellence-with-excellence match was deemed more appropriate. There were others who, while reluctantly accepting the scheme as proposed, questioned whether such a scheme would succeed with the South African PIs operating in HBUs. They argued that the scheme would best require identified South African PIs to relocate to HWUs.

## 3. The programme

**Figure 1.**
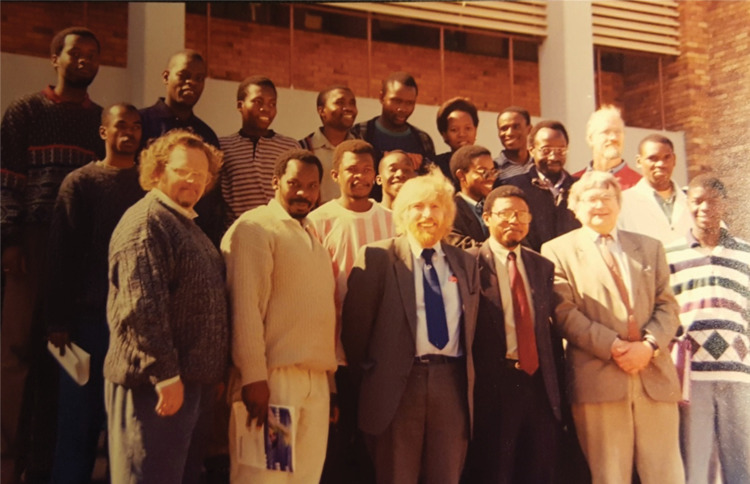
April 1996 visit by Richard Catlow and Alan Chadwick at the UNIN for preparation on commencement of the FRD/NRF–Royal Society collaboration, with staff and students of UNIN.

The collaboration officially started with RS–FRD funding in September 1996 and was based on several two-way planning visits over the previous 12 months; figure 1 recalls the successful visit of Richard Catlow and Alan Chadwick to UNIN in 1996.

The plan was for substantial (up to several weeks) exchange visits of researchers, particularly postgraduate students, and an annual assessment and planning meeting in the UNIN. As indicated in the Introduction, a strong scientific theme was established. The key aims were (i) the beneficiation of the local mining industries in South Africa, (ii) developing models of catalytic processes, (iii) designing novel materials for energy generation and storage, (iv) understanding the properties of precious metal alloys, and (v) experimental studies of the properties of key materials mentioned in (i)–(iv). The South African lead was Professor Phuti Ngoepe and his team at UNIN, which involved collaborations with groups at the Universities of Cape Town, Pretoria and Witwatersrand, along with CSIR, ESKOM and MINTEK. In the early years, aside from Phuti Ngoepe, the UNIN team of Happy Sithole, Reggie Kganyago, Thomas Netshisaulu, Peter Ntoahae, Michael Phala, Hasani Chauke and Regina Maphanga were relatively inexperienced; however, they quickly mastered the techniques and modelling procedures. Almost all completed doctoral theses, and now, many have professorial status. Although Phuti Ngoepe played a leading role in the collaboration at UL and was appointed as the Director of MMC, the first NRF–RS review of the project, in 1999, recommended the creation of Deputy Director and Administrator positions to support him and as a way of introducing succession planning. Indeed, those positions were created, and have been occupied by possible succession candidates, including Dr Lutz Ackermann and Professor Hasani Chauke.

From the outset of the programme, modelling expertise was to be primarily provided by the UK collaborators, and to this end, a team with very strong backgrounds was brought together, with Richard Catlow as the UK coordinator, who was then leading a large team in materials modelling at the Royal Institution in London. From the University of Oxford, Professor David Pettifor was recruited, who was a pioneer of computer modelling of metals using total energy density functional theory (DFT) calculations. David was also the founder of the materials modelling laboratory at Oxford, bringing together chemists, physicists and materials scientists, an especially useful asset to the programme. Professor Nick Quirke, then at Bangor University (now at Imperial College), brought expertise in molecular dynamics to the programme, and since 1986, he had been the (founding) editor of the *International Journal of Molecular Simulation*. Professor Saiful Islam, then at the University of Surrey (now at the University of Oxford), is an expert in molecular mechanics modelling, and he was an initial member of the UK team. Professor Kate Wright at Manchester University (now at the University of Western Australia) was in the initial team and had a background in minerals science that was essential for the projects involving mining problems. Professor Alan Chadwick (University of Kent) was in the team as an expert in experimental techniques to characterize solids. Soon after the start of the collaboration, four more members were recruited to the UK team. Professor Nicholas Harrison, also from Imperial College, had expertise in the application of DFT to materials modelling. At that time, DFT was relatively novel and has since become a very widely used method of studying the electronic structure of solids. Professor Steve Parker (University of Bath) gave the team a unique experience in the computer modelling of surfaces. Professor Nora de Leeuw at Birkbeck College (now at the University of Leeds) enhanced the expertise in minerals modelling. Dr Dean Sayle at Cranfield University, and later at the University of Kent, initiated the modelling of nanomaterials.

The successful format that was developed for the annual meetings in Limpopo was based on day talks by all the students involved in the collaboration, detailed discussions between UK supervisors and the students, an assessment and planning meeting of the teams and a concluding dinner in the Limpopo restaurant. The meetings attracted scientists from all parts of South Africa. One of notable attendees was Professor Frank Nabarro FRS (University of Witwatersrand) who was the most notable physicist in South Africa. He had pioneered the studies of the physics of solids and won numerous awards. He was responsible for Peierls–Nabarro stress and Nabarro–Herring creep, models of the mechanical behaviour of solids. He was an excellent contributor to the discussions following the talks by the students.

**Figure 2.**
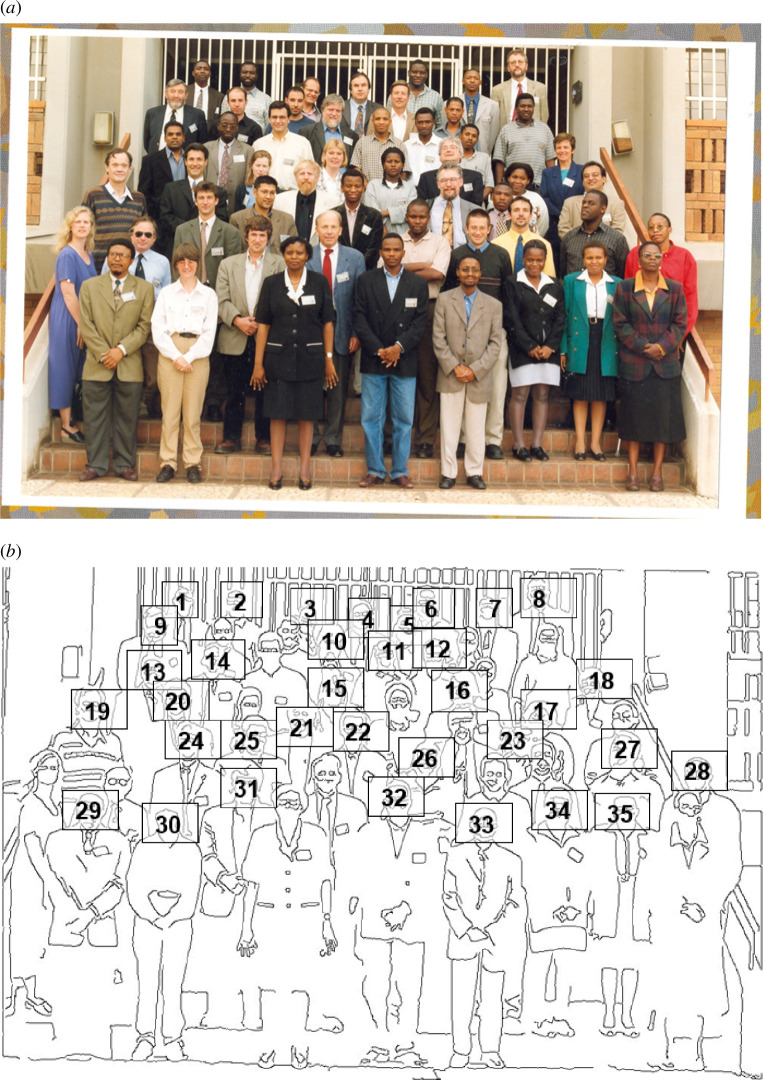
(*a*) Delegates of the Materials Modelling Meeting at Limpopo in 2000. (*b*) The names of the delegates in the photo. 1. Happy Sithole; 2. John Kudjoe; 3. Mario Valerio; 4. Rob Jackson; 5. Richard Williams; 6. Makonde Netsianda; 7. Maje Phasha; 8. Nick Quirke; 9. Shaun Corish; 10. Steve Parker; 11. Tjatji Tjebane; 12. Donald Mkhonto; 13. Nithaya Chetty; 14. Thomas Netshisaulu; 15. Nora de Leeuw; 16. Richard Catlow; 17 Sophy Maphalla; 18. Retha Rossouw; 19. Conrad Hartmann; 20. Mike Cortie; 21. Alan Chadwick; 22. Katse Maphoto; 23. Erasmus Rammutla; 24. Marc Meunier; 25. Saiful Islam; 26. Mike Phala; 27. Peter Ntoahae; 28. Leonard Segooa; 29. Phuti Ngoepe; 30. Lesley Cornish; 31 David Pettifor; 32. Jafta Dolo; 33 Khomotso Kganyago; 34. Regina Maphanga; 35. Dorah Masipa.

**Figure 3.**
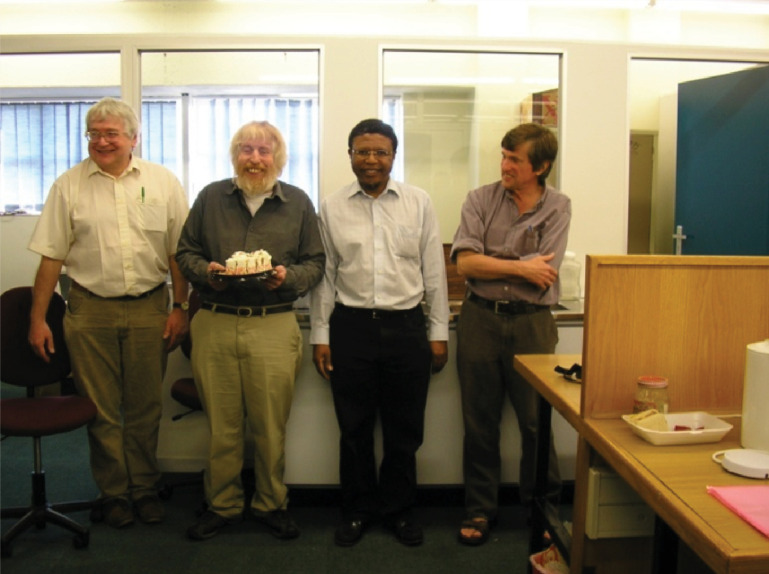
March 2007 visit by Richard Catlow, Alan Chadwick and David Pettifor for preparations on conclusion of the NRF–RS collaboration, appearing with Phuti Ngoepe in the photo—this coincided with Alan Chadwick’s 65th birthday.

The meetings always included a photograph of the participants, and the photo from 2000 is shown in figure 2, while figure 3 relates to a visit in 2007 to discuss the achievements of the programme and to plan future collaborations.

In the early stages of the collaboration, one of the major challenges was poor network connectivity, which made it difficult for collaborators at UL and the UK to connect remotely and share information. The problem was solved when UL was linked to a broad bandwidth by the South African National Research Network, charged with ensuring the successful participation of South African researchers in the global knowledge production endeavour.

Before discussing the outcomes of UNIN/UL programmes, we will briefly allude to other NRF–RS programmes conducted at other South African universities: a summary of these initiatives is provided below.

The NRF–RS programme at the UWC*,* led by Professor Jasper Rees, was the major driver in the expansion of the Biotechnology Department. UWC partnered with Universities of Oxford (Professor Ian Campbell), Cambridge (Professor Sir Tom Blundell) and Durham (Professors Toni Slabas and Keith Lindsay) with a focus on protein engineering and plant biotechnology and facilitated the training of a large number of postgraduate students and postdoctoral fellows. The current positions they are holding, both in South Africa and elsewhere in the continent, show the impact of the programme in developing highly successful researchers in biotechnology. The majority have remained in scientific careers and are based in Africa.

There was also a substantial impact of the NRF–RS programme at the University of Zululand’s Chemistry Department, and it was initially driven by the late Professor Mac Zulu, followed by Professor Gabriel Kolawole, with the support of the late Professor Paul O’Brien, initially, of Imperial College, and later, of Manchester University. A South African Research Chair Initiative (SARCHI) Chair, which was occupied by Professor Neeresh Revaprasadu (one of the programme recipients), has built up extensive knowledge and expertise in the field of nanomaterials synthesis during the past 20 years. Initially, the work focused on the use of molecular precursors for materials synthesis. Students were trained in various synthetic protocols and characterization techniques. During the past 5 years, there was a shift in the focus to the application of the novel materials in the field of energy generation and storage, an exciting and contemporary area of research. Several notable postgraduates and postdoctoral recipients of the programme are based at various institutions in and outside the country.

At the Department of Livestock and Pasture Science, UFH, under the leadership of Professor Jan Raats and with the support of the University of Edinburgh, the NRF–RS programme provided valuable assistance not only in research and training, but importantly, also towards the establishment of an extensive ecological monitoring programme in the 44 000 ha Great Fish River Reserve. The focus of this programme was on the long-term monitoring of the highly diversified vegetation and wildlife. At least 35 permanent transects with animal-friendly, yet elephant-proof concrete markers for bush and grass surveys were established and were monitored annually, which involved the undergraduate, postgraduate and staff members of UFH and collaborators.

An FRD–RS initiative grant was also given to the Department of Microbiology at the former *University of Durban-Westville* (UD-W—now University of Kwazulu Natal (UKZN)), in Durban, for the period from 1995 to 1999. Owing to internal institutional challenges, UD-W was unable to accept the grant, which was subsequently relocated in the UK and was later redirected to another South African institution. The PI at UD-W was Dr Dorsamy (Gansen) Pillay, with Professor Paul Broda from the University of Manchester Institute of Science and Technology as his counterpart. From 2000 to 2003, Dr Pillay subsequently received a South Africa/UK research grant for collaboration with researchers from the Scottish Crops Research Institute in Invergowrie, Dundee. The FRD–RS initiative served a catalytic role in advancing the South Africa–UK collaboration, as it impacted positively on other South African institutions in this collaboration, which included the University of Pretoria and the UWC. The outputs of this collaboration included publications and trained postgraduate students.

The NRF–RS thus embraced a broad range of science and resulted in an extensive capacity-strengthening of both institutions and people.

## 4. Outcomes

### 4.1. Scientific highlights

The Limpopo programme developed rapidly, with a growing focus on the fields of minerals, energy storage, alloys and polymers, which we will discuss in greater detail below.

#### 4.1.1. Minerals

Sulfide minerals are found in abundance in South Africa alongside platinum-rich compounds, such as PtS, PtAs_2_ and PtSb_2_, most of which are covalently bonded systems. Consequently, DFT methods were used to investigate how pressure affects the structural, electronic and optical properties of PtS, PtAs_2_ and PtSb_2_ as well as to calculate their elastic moduli [2,3]. Electronic and structural properties of pyrite FeS_2_ and marcasite obtained through *ab initio* calculations [4] have enabled the derivation of interatomic potentials for use in studies involving larger systems. Large quantities of cobalt, nickel and iron sulfide pentlandite compounds also occur as intergrowths with some of the platinum sulfides. The heats of formation obtained from DFT studies and the location of their Fermi levels relative to pseudo gaps in the electronic density of states substantiated why Co_9_S_8_ and Fe_4.5_Ni_4.5_S_8_ occur in nature, whereas Fe_9_S_8_ and Ni_9_S_8_ do not [5].

One of the major breakthroughs was the derivation of robust empirical interatomic potentials for pyrite, which could later be extended to accommodate other mineral sulfides. The potentials for FeS_2_ have predicted accurately the structural and elastic properties at ambient and high pressures [6] and high temperatures [7]. Studies employing similar interatomic potentials have also contributed to understanding the surface properties of the pyrite and marcasite forms of FeS_2_ [8]. Interatomic potentials were also derived for precious metal compounds, such as PtSb_2_ and PtAs_2_, where bulk and surface properties were predicted [9]. Results were obtained for precious metal sulfides with a tetragonal phase. The availability of potentials has provided an alternative way for calculating the bulk and surface properties of metal sulfides, such as ion transport, and point and line defects, flotation behaviour and dissolution. It also allows smelting, crystal growth and nanoparticle formation simulations involving up to hundreds of thousands of atoms considered in the same way as traditionally performed with metal oxides.

Ilmenite (FeTiO_3_) is abundant in South Africa, and it is beneficiated locally in the production of TiO_2_ pigments and titanium metal. A series of ilmenite-structured MeTiO_3_ compounds (Me = Fe, Mg, Zn and Mn) has been studied using DFT methods, where the structural and electronic properties were calculated in response to pressure [10]. However, DFT methods incorrectly modelled the highly correlated FeTiO_3_ as metallic instead of an insulator. A combined approach using the hybrid unrestricted Hartree–Fock and DFT methods correctly modelled the structure of FeTiO_3_, its electronic spin configuration and reproduced the experimental band gap of 3.0 eV [11].

Apatite plays a significant role as a biomaterial and as a mineral source of phosphate. The surface properties of the apatite structure have been studied [12]. Furthermore, the intergrowth of apatite and silica was investigated in detail as a precursor for work on bioglasses [13]. Vanadium dioxide (VO_2_), which forms rutile and monoclinic structures, has been investigated to understand its photochromic properties around the transition temperature of 68°C. Interatomic potentials were empirically derived to reproduce the low- and high-temperature phases (monoclinic and tetragonal, respectively) of VO_2_ as well as the monoclinic phase of tungsten dioxide. Tungsten dopants reduce the transition point towards room temperature [14].

#### 4.1.2. Energy storage

The initial work on energy devices focused on mixed-metal fluorites in the fast-ion phase for application in batteries. Static and molecular dynamics calculations and extended X-ray absorption fine structure spectroscopy (EXAFS) measurements were used to study how the temperature and composition of mixed-metal fluorites affect the defect and ion transport mechanisms [15,16]. Furthermore, light-scattering techniques and calculations on lanthanide-doped BaF_2_ and CaF_2_ provided insight into the changes in ion transport owing to various dopants [17,18]. LiC_6_ serves as an electrode for lithium batteries, and a comprehensive *ab initio* study on its structural and electronic properties [19], including associated voltage profiles [20], has been carried out. Magnesium-ion batteries are environmentally safe and offer higher energy densities when compared to traditional lithium-ion batteries [21]. The initial calculations on the magnesium-based, chevrel-structured MgMo_6_S_8_ were performed to predict its structure, energetics and voltage profiles for application as an electrode [22].

Electrolytic manganese dioxide (EMD) is a critical cathode material used in alkaline, lithium and sodium-ion batteries. The properties of EMD are reliant on its complex structure, and understanding that could help improve its properties. Surface energy calculations, molecular dynamics and EXAFS were used to study the intergrowths of pyrolusite and ramsdellite polymorphs [23]. Simulated amorphization and recrystallization techniques, involving tens of thousands of atoms, have been used to grow nanoparticles, bulk and nano-architectured forms comprising the intergrowths of these polymorphs [24–26]. The calculated radial distribution functions compare well with EXAFS results. These approaches have provided a wealth of information on the complex defects in EMD, including intergrowths, twinning, dislocations and vacancies.

#### 4.1.3. Metal alloys

Phase diagrams are important in the manufacturing of products, as they inform the process on which the thermodynamically stable phases of a material are applicable at different compositions, temperatures and pressures. DFT methods have been used to study the stability of precious metal superalloys, particularly Pt_3_Al, used in high-temperature jet engines. Competing phases, namely cubic L12 and tetragonal DOC structures in these superalloys, render them unsuitable for such applications. Tetragonal distortions and displacements which induce cubic to tetragonal transformation in Pt_3_Al have been identified. Electronic bands and density of states structures around the Fermi level were used to explain the causes of instability, and were compared with those of Ni_3_Al in which competing phases are not observed [27]. In addition, the Connolly–Williams methods have been used to study the cluster expansion of Pt_3_Al alloys and to determine the associated Hamiltonians, which enable the prediction of phase diagrams. PtAl_2_ is among the structures which appear in the platinum-modified bondcoats for nickel-based superalloys. The oxidation and sulfurization on PtAl_2_ surfaces have been investigated by DFT calculations, and the variation of reactivity of metals with alloying is predicted from electronic and structural properties [28] and agrees with available experimental results [29]. Studies of light metal alloys based on magnesium and aluminium were expanded, owing to their extensive use in the manufacturing of automotive components. The stabilization of a cubic phase of magnesium by alloying with lithium was investigated, which is more ductile than the hexagonal phase [30]. A model for predicting the phase stability and elastic properties of random alloys was proposed and applied to the binary Mg–Li system as a test study. The predicted phase stability trends at 0 K resemble those observed experimentally in the Mg–Li phase diagram, more especially at lower temperatures; and the method was found to be more accurate and less computationally demanding than the supercell approach [31]. Nanoparticles of gold are used for applications as catalysts. Since nanosystems require many atoms, so the semi-empirical Sutton–Chen potentials for gold have been refined to reproduce the surface energy and melting temperature of gold relatively accurately [32].

#### 4.1.4. Polymers

An understanding of the permeation by small gas molecules in polymers plays a vital role in enhancing the insulating properties of protective clothing. The effects of temperature, penetrant size and the nature of forcefields on penetrant (small molecules such as methane, helium and oxygen) diffusion and solubility in polysiloxanes have been investigated using molecular dynamics techniques and the Widom insertion method, respectively [33]. Further work involved studies of the transport of silicone oils in polysiloxanes networks, where the diffusion was well predicted [34]. Proton exchange membrane fuel cells use Nafion^®^ as an electrolyte; studies of diffusion and solubility of water and methanol in Nafion membranes were carried out, together with the impact of temperature on these transport properties [35].

In concluding this section, it is important to note the relevance of chosen collaboration themes to the geographical location of the university. Minerals and mineral processing were considered, owing to the large number of mines in the neighbourhood of UL and the Limpopo province. In the early stages of the collaboration, consideration was given to studying phosphates, which are related to agriculture; consequently, a paper was published on apatite, the main phosphorus-bearing mineral. However, owing to the large scope of the four identified themes, a fifth theme was not pursued, but this could be considered in the future.

### 4.2. People

Over the years several physics and chemistry honours students have done research projects in the MMC and they have been feeders to postgraduate studies at both the MMC and other institutions. MSc and PhD students and postdoctoral researchers have progressed to the positions of lecturers and researchers at universities, including the North/Limpopo, South Africa, Venda, Fort Hare, Pretoria, Witwatersrand, the Council for Scientific and Industrial Research, Mintek, Transnet, Johnson and Matthey, in both South Africa and the UK, DeBeers and government departments, including the Centre for High Performance Computing (CHPC) and the National Integrated Cyber Infrastructure Systems (NICIS). As examples, Dr Happy Sithole—the current Centre Manager of NICIS; Professor Hasani Chauke—the current Director of the School of Physical and Mineral Sciences (UL); Dr Regina Maphanga—CSIR; Dr Maje Phasha—Mintek; and Dr Helen Chuma—Johnson and Matthey, UK, are some of the direct and indirect beneficiaries of the NRF–RS programme. Some of the excellence indicators are MSc distinction passes and many prizes that were awarded for notable presentations at national and international conferences by such students, from 2000 to date. A significant number of the graduates are women, who have, in addition, played a significant role in the exposure of women to the sciences in South Africa, such as Women in High Performance Computing and Women in Physics South Africa.

In addition, the MMC has produced unique expertise on computing systems management, who have helped the university in reducing maintenance costs and some have proceeded to organizations such as the CHPC. Given that the MMC’s operations are primarily rooted in research and development, it is essential for the systems managers to engage in active research in both computational modelling of materials and high-performance computing (HPC). The responsibilities of systems managers cover user support and training and HPC systems management. Their knowledge base also ranges over software applications, data management, operating systems, virtualization, storage and networking. The scope of their responsibilities includes reviewing ongoing features and advances made in software and hardware technologies. They analyse how well computing systems fit into MMC’s operations and monitor their effectiveness and conduct upgrades when necessary. In addition to involvement in postgraduate supervision, they also teach and train undergraduate students in HPC cluster building. In 2018, one of the trained students was a member of Team SA that secured a third place in cluster building at the International Supercomputing Conference in Frankfurt, Germany.

### 4.3. Institutions

The collaboration helped to strengthen institutional linkages between South Africa and the UK, but more importantly, it assisted in building not only the new centre but also sustained further collaboration around modelling and simulation. One such institution is the formation of the CHPC in South Africa. During the 1990s, South Africa did not have any form of national HPC facility, and thus with small clusters at the UNIN, initial computations were achieved, but the work depended heavily on the UK systems for scaling and refining the computations, which helped to convince the South African government that there is a strong business case for investing in HPC facilities, as the value of higher scale computations was clearly visible.

The process to establish the CHPC started with identifying talent within the country to lead and operate such a facility, and Happy Sithole and Khomotso Kganyago, who were the beneficiaries of this collaboration, who gained exposure to HPC through this initiative, were tasked with this responsibility by the South African Departments of Science and Technology. The UK collaborators provided significant input in the formation and sustenance of the CHPC, with the following examples.

— *Strategic aspects*: Richard Catlow, Alan Chadwick, David Pettifor and Nick Quirke helped with formulating institutional arrangements for ensuring input from communities in operating and provisioning such systems, based on their experience with the UK communities. These helped in developing flagship projects and special interest Groups that formed the ecosystem of CHPC.
— *User applications and research facilitation* was another important aspect of building a successful and sustainable HPC operation, and users from the UK, with their in-depth knowledge of applications, assisted in this area, and they were led by Martyn Guest (now at Cardiff University) in sharing the knowledge in developing benchmarks for the HPC systems and selecting a representative applications suite to help get a balanced system. The team from Daresbury Laboratories and Michael Payne’s group (in Cambridge) provided critical input that helped South Africa to acquire systems useable by most of the science and engineering applications.
— *Strategic HPC deployments and acquisition*: Another critical area where the UK team assisted and continue to do so is through the collaboration with Paul Calleja from Cambridge, Adrian Wander and Scott Woodley. Through this collaboration, important strategies were developed in optimizing the acquisition and also forming strategic partnerships with original equipment manufacturers, which enabled South Africa to build systems and get them on the TOP 500 list with minimal budget, because the teams worked together to get equipment and deploy systems themselves.

**Figure 4.**
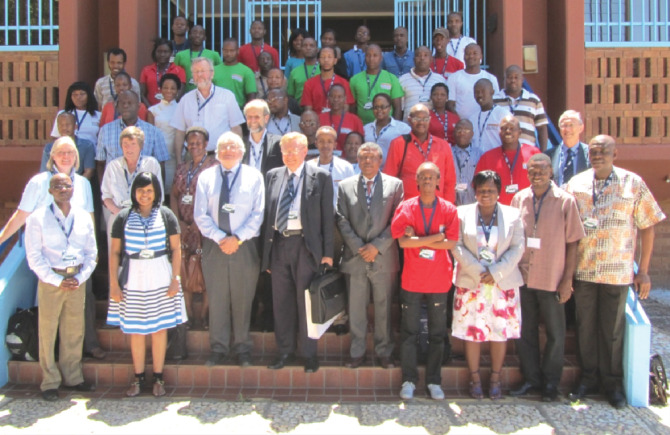
Delegates at a conference to mark Phuti Ngoepe’s 60th birthday: 8 and 9 January 2013, at the UL.

These collaborations continue and produce high-impact projects in HPC, including linkages with South African Weather Services (SAWS) and the UK Met Office, which provides operational forecasting for the entire country. The three-way collaboration between CHPC, UK Met Office and SAWS is the heart of the success of weather forecasting in the country. Other strategic developments are in the areas of collaborative computational projects (CCPs) and National Institute of Theoretical Computational Science, where the collaboration between UK and South Africa continues to provide guidance in building communities that respond to fast changing technological advances and strengthen modelling and simulation. The collaboration is not limited to South Africa, but has managed to broaden to other African countries, as demonstrated by building the capacity in other African countries by providing HPC systems, through a Cambridge University donation and collaboration in materials science—helped by Richard Catlow and Nora De Leeuw. Today, successful applications from other African countries come from the Kenya, Ghana and Namibia collaborators in materials science and quantum chemistry. Most of the SADC countries have small HPC systems, and with the partnership, the Cambridge team and South Africa helped build and operate the HPC system in Morocco. The collaboration is also further strengthened by the participation in meetings, such as the Annual HPC Meeting taking place in South Africa, where the UK collaborators continue to provide keynote addresses and workshops alongside the conference, in areas of material science. Similarly, South Africa collaborators continue to participate in the meetings in the UK of the CCP5 Annual General Meetings and also to celebrate collaborator milestones, for example, the symposium in celebration of Phuti Ngoepe’s 60th birthday held in 2013 (figure 4).

## 5. Current programme

Beyond the formal NRF–RS collaboration, themes that were developed have continued under various local and international programmes, some of the latter with the UK team, with an emphasis on industrial applications. Two major successful developments, in 2007, emanated from the Departmenet of Science and Technology–NRF SARCHI allocated to Phuti Ngoepe, which ran for 15 years (2007–2021). The MMC has now been formally incorporated in the university structure, where full-time staff members have been allocated. The second is the access to the CHPC, which was also launched in 2007, where MMC was allocated one of the first three flagship projects, and the CHPC has since been used for computations. The next section gives a synopsis of the highlights of the current programme.

### 5.1. Research focus areas

#### 5.1.1. Mineral processing

After studies on minerals under the NRF–RS programme, the emphasis shifted to mineral processing, which emanated from a collaboration with Anglo American Platinum and the creation of a cooperative effort, South African Minerals to Metals Research Initiative (SAMMRI), between the mining companies and universities. The focus was on efficient mineral processing methods to address the challenges of water, energy and environmental conservation in the mining sector, which incorporated fundamental research. The MMC has since studied the oxidation and hydration of metal sulfides hosting base and precious metals [36], and it has developed proofs of concept for designing reagents that can efficiently recover minerals from ores in collaboration with the University of Cape Town and Beijing General Research Institute in Mining and Minerals (BGRIMM) [37,38].

#### 5.1.2. Energy storage

Previous studies on energy materials evolved to high-energy-density batteries that are central to the development of electric vehicles, solar energy storage and electricity utility backups and are key to climate change interventions, partly under the Department of Science and Innovation (DSI) Energy Storage RDI Consortium. Their enhanced performance is now achieved with nano-architectured electrodes, including primary nanoparticles. The MMC has applied some of the world-leading simulation approaches to produce such nanostructures in Li-ion batteries [39,40] and first principles methods [41], which shed valuable insights on precursor reactor processes occurring in laboratories, industrial pilot and production plants. It has also applied machine learning cluster expansion methods to determine the stability of doped NMC cathodes [42] and has carried out surface calculations for cathodes [43] and metal air batteries [44,45].

#### 5.1.3. Alloy development

Based on the emphasis on precious metal alloys in the NRF–RS programme [27], the phase stabilities of precious and light metal alloys have been studied using a combination of energetics, elastic properties and phonon dispersions at the DSI Titanium Competency Centre. This approach has provided valuable information for shape memory devices, and generally, powder metallurgy processing [46,47]. Titanium cluster simulation studies conducted at the pilot plant facility at the CSIR have given valuable insights on titanium metal growth [48]. Recent studies have been extended to magnets [49], including where machine learning cluster expansion methods were employed to find ductile high saturation magnetization and Curie temperature alloys [50].

#### 5.1.4. Experimental

Recently, a synthesis laboratory for cathode materials employing the co-precipitation method has been established at the university [51], which, in addition to growing such materials synthetically, can grow such compounds from raw materials, which is linked to a precursor pilot plant in Mbombela. The facility has been complemented by the ability of the MMC to predict stable phases on the doping of cathode materials using machine learning cluster expansion methods; and characterization methods are available at other South African laboratories and initiatives such as the UK Synchrotron Techniques for African Research and Technology.

#### 5.1.5. Piloting

Over the years, the MMC’s research activities have been guided by and integrated into industrial initiatives. The Centre was instrumental in the retention and development of a pilot plant for manufacturing cathode manganese-based precursors for Li-ion batteries (now called Cathode Materials Project), in Mbombela, South Africa, which plays a critical role in the production of battery components. The Centre’s simulation work on titanium clusters has given valuable insights to the titanium metal growth and pilot plant facility at the CSIR. In collaboration with SAMMRI in South Africa and BGRIMM in China, the Centre engaged, through simulations and experiment, in the design of flotation reagents that tend to enhance the recovery of base and precious metals from the ore in the mines.

#### 5.1.6. Some outputs

To date, MMC has trained to completion many honours students, 34 PhDs and 56 MScs, and it has contributed several publications, book chapters and presentations at national and international conferences, including invited contributions. It has hosted several local and international conferences that were aligned with its research themes. In addition, MMC mentored HPC systems managers, with PhDs, who have saved the institution significant maintenance costs and worked at other HPC centres. Between 2000 and 2023, the MSc and PhD students and research associates have received over 80 awards at institutional, national and international conferences and occasions. Different organizations and forums have bestowed honours on Professor Ngoepe for various contributions related to his scientific field, capacity-building and service to community, including the prestigious Order of Mapungubwe award by the President of the Republic of South Africa, partly for building the Centre at UL, and the Chinese Government Friendship Award.

## 6. Conclusions

The UK–Limpopo collaboration clearly achieved its aims of both promoting the collaboration between South African and UK scientists in the field of materials modelling and establishing a centre of excellence at Limpopo, which is successful and self-sustaining and continues to have a broad impact in terms of both the scientific output and the training of scientists in a key area of contemporary science and technology. Moreover, the collaboration has led to broader lessons regarding successful ‘North–South’ collaborations which we now consider.

The first is that such collaborations must be partnerships of equals: both have expertise and approaches to learn from their partners; and the two-way approach should apply to people. Indeed, in this collaboration, UK students, postdocs and more senior staff spent substantial periods working in the Limpopo centre and there were many successful visits of UL staff and students to the UK. Secondly, these types of collaboration need time to develop: if the RS–NRF collaboration had ended after 4 or 5 years, although there would have been undoubted successes, the centre would probably have not been sufficiently mature to have achieved long-term sustainability; continuing and consistent support is needed for these programmes to achieve their goals. Thirdly, although ‘capacity-strengthening’ programmes are usually justified and judged on their efficacy in training and institutional building, they often also lead to original and novel science of high quality and to science which would not have happened otherwise. Some examples of the novel science pertain to modelling metal oxide nano-architectures, such as nanoparticles, nanosheets, nanorods and nanoporous structures, through the simulated amorphization recrystallization method that was initially developed by Dean Sayle [52]. In the collaboration, the approach was adapted to energy storage materials, and it currently continues to play a significant role in their synthesis and performance evaluation [24,40,53–55]. Another of the many highlights involved usage of a hybrid-exchange density functional approach to study highly correlated systems, including minerals like ilmenite, which demonstrated reliable and predictive simulations of structure and properties [11]. Furthermore, an excellent foundation was laid in applications of cluster expansion methods and Monte Carlo simulations to alloys [56], which is currently used routinely towards the production of phase diagrams for materials, in general [57]. Finally, studies on DFT calculations for base and precious metal sulfides [2,4,58], and development of interatomic potentials for atomistic simulations [6–9], are noteworthy. These provided a basis for current investigations on the recovery of minerals, including proof of concept depicting good correlation between modelling and experimental results [37,38]. In addition, simplified procedures for predicting the stabilities of base metal pentlandite structures were formulated [5] and have been recently extended to precious metal pentlandites [59].

Finally, it is worth emphasizing that the collaboration was an enriching and enjoyable experience for all involved.

## Data Availability

This article has no additional data.

## References

[1] Catlow CRA, De Leeuw NH, Michaelides A, Woodley SM. 2023 Supercomputing modelling of advanced materials: preface. Phil. Trans. R. Soc. A **381**, 20220252. (10.1098/rsta.2022.0252) 37211036 PMC10200345

[2] Nguyen-Manh D, Ntoahae PS, Pettifor DG, Ngoepe PE. 1999 Electronic structure of platinum-group minerals: prediction of semiconductor band gaps. Mol. Simul. **22**, 23–30. (10.1080/08927029908022083)

[3] Mangwejane SS. 2005 Atomistic, electronic and optical studies of Ptsb_2_ and Ptbi_2_. MSc dissertation, University of the North, Sovenga, South Africa.

[4] Nguyen-Manh D, Pettifor DG, Sithole HM, Ngoepe PE, Arcangeli C, Tank R, Jepsen O. 1997 Electronic structure, pressure dependence and optical properties of FeS_2_. MRS Proc. **491**, 401–406. (10.1557/PROC-491-401)

[5] Chauke HR, Nguyen-Mahn D, Ngoepe PE, Pettifor DG, Fries SG. 2002 Electronic structure and stability of pentlandites: Co_9_S_8_ and the related alloys. Phys. Rev. B **66**, 155105. (10.1103/PhysRevB.66.155105)

[6] Sithole HM, Ngoepe PE, Wright K. 2003 Atomistic simulation of the structure and elastic properties of pyrite (FeS_2_) as a function of pressure. Phys. Chem. Miner. **30**, 615–619. (10.1007/s00269-003-0359-6)

[7] Tlali SB, Mathe BA, Kotane LM, Schöning FRL, Comins JD, Every AG, Sithole HM, Ngoepe PE, Wright KV. 2004 Brillouin scattering studies and computational simulations of the elastic properties of pyrite (FeS_2_) at high temperatures. Phys. Stat. Sol. **1**, 3073–3076. (10.1002/pssc.200405378)

[8] de Leeuw NH, Sithole HM, Parker SC, Ngoepe PE. 2000 Modelling surface stability and reactivity of pyrite: introduction of the new potential model. J. Phys. Chem. **104**, 7969–7976. (10.1021/jp0009498)

[9] Ngoepe PE, Ntoahae PS, Mangwejane SS, Sithole HM, Parker SC, Wright KV, de Leeuw NH. 2005 Atomistic simulation studies of iron sulphide, platinum antimonide and platinum arsenide. S. Afr. J. Sci. **101**, 480–483. http://hdl.handle.net/10520/EJC96441

[10] Mkhonto D. 2002 Computer simulation of ilmenite systems. MSc dissertation, University of the North, Sovenga, South Africa.

[11] Wilson NC, Muscat J, Mkhonto D, Ngoepe PE, Harrison NM. 2005 Structure and properties of ilmenite from first principles. Phys. Rev. B **71**, 075202. (10.1103/PhysRevB.71.075202)

[12] Mkhonto D, de Leeuw NH. 2002 A computer modelling study of the effect of water on the surface structure and morphology of fluorapatite: introducing a Ca_10_(PO_4_)_6_F_2_ potential model. J. Mater. Chem. **12**, 2633–2642. (10.1039/b204111a)

[13] de Leeuw NH, Mkhonto D, Catlow CRA. 2003 A computer modeling study of the adhesion of apatite thin films on silicate surfaces. J. Phys. Chem. B **107**, 1–3. (10.1021/jp026757p)

[14] Netsianda M, Ngoepe PE, Catlow CRA, Woodley SM. 2008 The displacive phase transition of vanadium dioxide and the effect of doping with tungsten. Chem. Mater. **20**, 1764–1772. (10.1021/cm701861z)

[15] Netshisaulu TT, Ngoepe PE, Chadwick AV. 1999 Computer modelling and EXAFS study of disorder in CdF_2_(xPbF_2_) mixed systems. Mol. Simul. **22**, 1–21. (10.1080/08927029908022082)

[16] Netshisaulu TT, Chadwick AV, Ngoepe PE, Catlow CRA. 2005 Spectroscopic and computer modeling studies of lead-cadmium fluoride. J. Phys. Condens. Matter **17**, 6575–6586. (10.1088/0953-8984/17/41/026)

[17] Rammutla KE, Comins JD, Erasmus RM, Netshisaulu TT, Ngoepe PE, Chadwick AV. 2002 Investigation of the superionic behaviour of BaF_2_ (x mol% LaF_3_) by Raman and Brillouin scattering and molecular dynamics simulations. Radiat. Eff. Def. Solids **157**, 783–788. (10.1080/10420150215788)

[18] Mujaji M, Mjwara PM, Comins JD, Rammutla KE, Ngoepe PE. 2005 Light scattering and computational simulations of the superionic behaviour of CaF_2_ doped with lanthanide ions. Phys. Stat. Sol. **2**, 490–494. (10.1002/pssc.200460215)

[19] Kganyago KR, Ngoepe PE. 2003 Structural and electronic properties of graphite and lithium intercalated graphite. Phys. Rev. B **68**, 205111. (10.1103/PhysRevB.68.205111)

[20] Kganyago KR, Ngoepe PE, Catlow CRA. 2003 Ab initio calculation of the voltage profile for LiC_6_. Solid State Ion. **159**, 21–23. (10.1016/S0167-2738(02)00763-4)

[21] Aurbach D, Lu Z, Schechter A, Gofer Y, Gizbar H, Turgeman R, Cohen Y, Moshkovich M, Levi E. 2000 Prototype systems for rechargeable magnesium batteries. Nature **407**, 724–727. (10.1038/35037553) 11048714

[22] Kganyago KR, Ngoepe PE, Catlow CRA. 2003 Voltage profile, structural prediction and electronic calculations for Mg_x_Mo_6_S_8_. Phys. Rev. B **67**, 104103. (10.1103/PhysRevB.67.104103)

[23] Maphanga RR, Ngoepe PE, Parker SC, Chadwick AV. 2003 Computational modeling and EXAFS studies of electrolytic manganese dioxide. In Proc. 2nd Int. Conf. of the African Materials Research Society, Johannesburg, South Africa, 8–11 December 2003.

[24] Sayle TXT, Catlow CRA, Maphanga RR, Ngoepe PE, Sayle DC. 2005 Generating MnO₂ nanoparticles using simulated amorphization and recrystallization. J. Am. Chem. Soc. **127**, 12828–12837. (10.1021/ja0434073) 16159276

[25] Sayle TXT, Catlow CRA, Maphanga RR, Ngoepe PE, Sayle DC. 2006 Atomistic models for MnO_2_ generated using amorphisation and recrystallization. J. Cryst. Growth **294**, 118–129. (10.1016/j.jcrysgro.2006.05.033)

[26] Sayle TXT, Maphanga RR, Ngoepe PE, Sayle DC. 2009 Predicting the electrochemical properties of MnO_2_ nanomaterials used in rechargeable Li batteries: simulating nanostructure at the atomistic level. J. Am. Chem. Soc. **131**, 6161–6173. (10.1021/ja8082335) 19206514

[27] Chauke HR, Minisini B, Drautz R, Nguyen-Manh D, Ngoepe PE, Pettifor DG. 2010 Theoretical investigation of the Pt_3_Al ground state. Intermetallics **18**, 417–421. (10.1016/j.intermet.2009.08.016)

[28] Mokagane MM, Ngoepe PE, Harrison NM, Montanari B. 2003 Adsorption of sulphur on PtAl₂. In Proc. 2nd Int. Conf. of the African Materials Research Society, Johannesburg, South Africa, 8–11 Decomver 2003.

[29] Rodriguez JA, Kuhn M. 1997 Interactions between sulfur and platinum in bimetallic surfaces: reaction of S₂ with Pt–Al alloys. J. Vac. Sci. Technol. A **15**, 1608–1612. (10.1116/1.580640)

[30] Phasha MJ. 2005 Ab Initio study of cohesive, electronic and elastic properties of ordered cubic-based mg–Li alloys. MSc thesis, University of the North, Sovenga, South Africa.

[31] Phasha MJ, Ngoepe PE. 2012 An alternative DFT-based model for calculating structural and elastic properties of random binary HCP, FCC and BCC alloys: Mg–Li system as test case. Intermetallics **21**, 88–96. (10.1016/j.intermet.2011.09.015)

[32] Mahladisa MA, Ackermann L, Ngoepe PE. 2005 Structural properties of gold clusters at different temperatures. S. Afr. J. Sci. **101**, 471–474.

[33] Segooa LRM, Ngoepe PE, Goldbeck-Wood G. 2001 Atomistic simulation of gas phase atoms with RADII through polysiloxane. Radiat. Eff. Def. Solids **156**, 341–346. (10.1080/10420150108216915)

[34] Kubai T. 2007 Computer modelling studies of the diffusion of low molecular weight cyclic PDMS oligomer in PDMS polymer. MSc thesis, University of Limpopo, Sovenga, South Africa.

[35] Dolo JJ, Ackermann L, Ngoepe PE. 2003 Molecular dynamics simulation studies of water and methanol in Nafion membrane. In Proc. 2nd Int. Conf. of the African Materials Research Society, Johannesburg, South Africa, 8–11 December 2003.

[36] Mkhonto PP, Ngoepe PE, Chauke HR. 2015 Ab initio studies of O_2_ adsorption on (110) nickel-rich pentlandite (Fe_4_Ni_5_S_8_) mineral surface. Minerals **5**, 665–678. (10.3390/min5040516)

[37] Mkhonto PP, Zhang X, Lu L, Xiong W, Zhu Y, Han L, Ngoepe PE. 2022 Adsorption mechanisms and effects of thiocarbamate collectors in the separation of chalcopyrite from pyrite minerals: DFT and experimental studies. Miner. Eng. **176**, 1–14. (10.1016/j.mineng.2021.107318)

[38] McFadzean Belinda, Mkhonto P, Ngoepe PE. 2023 Interactions of xanthates of increasing chain length with pyrite surfaces: a DFT-D and microcalorimetry study. Appl. Surf. Sci. **607**, 154910. (10.1016/j.apsusc.2022.154910)

[39] Matshaba MG, Sayle DC, Sayle TXT, Ngoepe PE. 2016 Structure of surface entrance sites for Li intercalation into TiO_2_ nanoparticles, nanosheets, and mesoporous architectures with application for Li-ion batteries. J. Phys. Chem. C **120**, 14001–14008. (10.1021/acs.jpcc.6b04770)

[40] Ledwaba RS, Sayle DC, Ngoepe PE. 2020 Atomistic simulation and characterisation of spinel Li_1+*x*_Mn_2_O_4_ (0 ≤ x ≤ 1) nanoparticles. ACS Appl. Energy Mater. **3**, 1429–1438. (10.1021/acsaem.9b01870)

[41] Morukuladi MT, Lethole NL, Masedi MC, Ngoepe NN, Ngoepe PE. 2022 Structural, electronic, elastic and dynamical properties of MCO_3_ (M: Mn, Co, Ni) precursor materials for Li-ion batteries: a first-principles study. J. Electrochem. Soc. **169**, 020540. (10.1149/1945-7111/ac50e3)

[42] Mphahlele MG, Masedi MC, Ledwaba RS. 2023 The effect of Ni-doping on the stability of Li_2_MnO_3_ cathode material: a DFT study. MATEC Web Conf. **388**, 07005. (10.1051/matecconf/202338807005)

[43] Ramogayana B, Santos-Carballal D, Maenetja KP, Malatji KT, de Leeuw NH, Ngoepe PE. 2022 A DFT+U-D3 study of the adsorption of hydrogen fluoride and ethylene carbonate on the niobium-doped (001), (011) and (111) surfaces of lithium manganese oxide. J. Electrochem. Soc. **169**, 090507. (10.1149/1945-7111/ac8e35)

[44] Mellan TA, Maenetja KP, Ngoepe PE, Woodley SM, Catlow CRA, Grau-Crespo R. 2013 Lithium and oxygen adsorption at the β-MnO_2_ (110) surface. J. Mater. Chem. A **1**, 14879–14887. (10.1039/c3ta13559d)

[45] Maenetja KP, Ngoepe PE. 2022 Unravelling catalytic activity of MnO₂, TiO₂ and VO₂ (110) surfaces by oxygen coadsorption on sodium-adsorbed MO₂ {M = Mn, Ti, V}. ACS Omega **7**, 25991–25998. (10.1021/acsomega.1c05990) 35936399 PMC9352334

[46] Mahlangu R, Phasha MJ, Chauke HR, Ngoepe PE. 2013 Structural, elastic and electronic properties of equiatomic PtTi as potential high-temperature shape memory alloy. Intermetallics **33**, 27–32. (10.1016/j.intermet.2012.09.021)

[47] Mashamaite MP, Chauke HR, Ngoepe PE. 2019 The effects of Ru, Cu, Zr and Hf on mechanical properties in Ti-Pt high temperature shape memory alloys. IOP Conf. Ser. Mater. Sci. Eng. **655**, 012011. (10.1088/1757-899X/655/1/012011)

[48] Lazaruskas T *et al*. 2018 Thermodynamically accessible titanium clusters Ti_N_, N = 2–32. Phys. Chem. Chem. Phys. **20**, 13962–13972. (10.1039/C8CP00406D) 29744486

[49] Diale RG, Ngoepe PE, Moema JS, Phasha MJ, Moller H, Chauke HR. 2023 A computational study of the thermodynamic and magnetic properties of Co-alloyed MnPt. MRS Adv. **8**, 651–655. (10.1557/s43580-023-00568-4)

[50] Ledwaba TM, Diale RG, Ngoepe PE, Chauke HR. 2022 Structural and stability of B2 FeCo1-XVX and Fe1-XCoVX systems: cluster expansion approach. SAJST **40**, 98–101. (10.36303/SATNT.2021cosaami.19)

[51] Ngoepe N, Gutierrez A, Barai P, Chen J, Ngoepe PE, Croy JR. 2021 The effects of process parameters on the properties of manganese-rich carbonate precursors: a study of co-precipitation synthesis using semi-batch reactors. Chem. Eng. Sci. **241**, 1–11. (10.1016/j.ces.2021.116694)

[52] Ngoepe PE, Maphanga RR, Sayle DC. 2013 Toward the nanoscale. In Computational approaches to energy materials (eds CRA Catlow, A Sokol, A Walsch), pp. 261–290. Chichester, UK: John Wiley & Sons Ltd. (10.1002/9781118551462.ch9)

[53] Sayle TXT, Kgatwane K, Ngoepe PE, Sayle DC. 2016 ‘Breathing-crystals’ the origin of electrochemical activity of mesoporous Li–MnO_2_. J. Mater. Chem. A **4**, 6456–6464. (10.1039/C6TA01832G)

[54] Ledwaba RS, Kgatwane KM, Sayle DC, Ngoepe PE. 2022 Structural characterisation and mechanical properties of nanosized spinel LiMn₂O₄ cathode investigated using atomistic simulation. Mater. Res. Bull. **146**, 111611. (10.1016/j.materresbull.2021.111611)

[55] Ledwaba RS, Tsebesebe NT, Ngoepe PE. 2022 Unraveling the role of oxygen and manganese charge compensation during nucleation and crystal growth of Li-rich layered Li_1.2_Mn_0.8_O_2_ cathode materials. J. Electrochem. Soc. **169**, 110502. (10.1149/1945-7111/ac9d06)

[56] Chauke HR. 2006 First-principles approach: energetics and phase stability modelling of PT/PT_3_AI alloys. PhD thesis, University of Limpopo, Sovenga, South Africa.

[57] Maphoto RI, Morukuladi MT, Malatj KT. 2022 First-principle study of CsPbBr_3_ and CsPbI_3_ perovskite solar cells. ECS J. Solid State Sci. Technol. **11**, 035012. (10.1149/2162-8777/ac5eb6)

[58] Marmier A, Ntoahae PS, Ngoepe PE, Pettifor DG, Parker SC. 2010 Negative compressibility in platinum sulfide using density-functional theory. Phys. Rev. B **81**, 172102. (10.1103/PhysRevB.81.172102)

[59] Molala KB, Mkhonto PP, Mehlape MA, Ngoepe PE. 2023 First principles study on stability of base and precious metals pentlandite-like compounds. Theor. Chem. Acc. **142**, 6. (10.1007/s00214-022-02951-0)

